# Neuronal Activity in Rat Barrel Cortex Underlying Texture Discrimination 

**DOI:** 10.1371/journal.pbio.0050305

**Published:** 2007-11-13

**Authors:** Moritz von Heimendahl, Pavel M Itskov, Ehsan Arabzadeh, Mathew E Diamond

**Affiliations:** 1 Cognitive Neuroscience Sector, International School for Advanced Studies, Trieste, Italy; 2 SISSA Unit, Italian Institute of Technology, Trieste, Italy; 3 School of Psychology, University of Sydney, Sydney, Australia; Vanderbilt University, United States of America

## Abstract

Rats and mice palpate objects with their whiskers to generate tactile sensations. This form of active sensing endows the animals with the capacity for fast and accurate texture discrimination. The present work is aimed at understanding the nature of the underlying cortical signals. We recorded neuronal activity from barrel cortex while rats used their whiskers to discriminate between rough and smooth textures. On whisker contact with either texture, firing rate increased by a factor of two to ten. Average firing rate was significantly higher for rough than for smooth textures, and we therefore propose firing rate as the fundamental coding mechanism. The rat, however, cannot take an average across trials, but must make an immediate decision using the signals generated on each trial. To estimate single-trial signals, we calculated the mutual information between stimulus and firing rate in the time window leading to the rat's observed choice. Activity during the last 75 ms before choice transmitted the most informative signal; in this window, neuronal clusters carried, on average, 0.03 bits of information about the stimulus on trials in which the rat's behavioral response was correct. To understand how cortical activity guides behavior, we examined responses in incorrect trials and found that, in contrast to correct trials, neuronal firing rate was higher for smooth than for rough textures. Analysis of high-speed films suggested that the inappropriate signal on incorrect trials was due, at least in part, to nonoptimal whisker contact. In conclusion, these data suggest that barrel cortex firing rate on each trial leads directly to the animal's judgment of texture.

## Author Summary

How cortical activity contributes to sensation is among biology's oldest problems. We studied the nature of the cortical representations underlying judgments of texture in rats. The rodent whisker sensory system is particularly intriguing because it is “active”: the animal generates sensory signals by palpating objects through self-controlled whisker motion (just as we move our fingertips along surfaces to measure their tactile features). Rats touched rough or smooth textures with their whiskers and turned left or right for a reward according to the texture identity. Monitoring behavior with high-speed videography, we have found that on trials when the rat correctly identified the stimulus, the firing rate of cortical neurons varies during a window of a few hundred milliseconds before making a decision according to the contacted texture: high for rough and lower for smooth. This firing-rate code is reversed on error trials (lower for rough than smooth). So when cortical neurons report the wrong stimulus, the rat, “feeling” the signals of its cortical neurons, fails to identify the stimulus. We conclude that barrel cortex firing rate on each trial predicts the animal's judgment of texture. This experiment begins to elucidate which features of cortical activity underlie the animal's capacity for tactile sensory discrimination.

## Abstract 3

Rats palpate objects with their whiskers to perceive texture. Their judgment of texture is predicted by the firing rate of neurons in the somatosensory cortex.

## Introduction

One aim in studies of sensory coding is to quantify how neuronal activity represents objects in the external world. In rats, as in humans [1], tactile exploration entails the interplay of motor output and sensory input: Rats palpate objects by sweeping their whiskers in a rhythmic forward–backward cycle [2]. This active sensing gives rise to a number of well-developed tactile capacities [3–6], including the sense of texture [4]. The aim of the present work was to explore the neuronal coding of textures in rats while they perform a discrimination task.

The signals from each whisker reach layer IV “barrels” of primary somatosensory cortex [7] after synaptic relays through the brain stem and thalamus. In the barrel cortex of anesthetized rats, the whisker vibrations associated with different textures evoke cortical responses that differ according to texture—coarser textures evoke more spikes per sweep [8,9]. By extending this line of investigation to awake rats, we now ask which features of sensory coding are conserved during active exploration of the environment, when stimuli are not imposed on the receptors, but are generated by the animal through its own motor program. Because the behaving animal makes choices based on the signals carried by its sensory neurons, we can ask how the neuronal code leads to the animal's decisions.

## Results

### Texture Discrimination Task and Cortical Spike Trains

**Figure 1:**
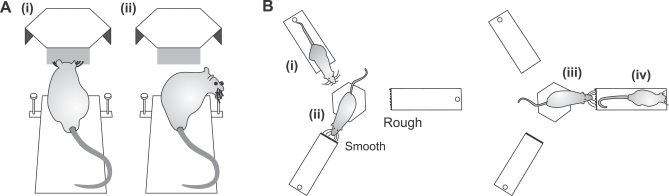
Texture Discrimination Tasks Trials sketched from camera images. (A) The 1-arm task. (i) The rat perched on the edge of the platform and extended to touch the rough texture (gray rectangle) with its whiskers. (ii) Having identified the texture, the rat turned to the right drinking spout and received a water reward. (B) The 3-arm task. (i) The rat began by crossing from the start arm to the central platform, (ii) touched the smooth-textured discriminandum with its whiskers, (iii) rejected the first texture and proceeded to touch the rough-textured discriminandum, and (iv) crossed the gap to collect a water reward at the distant part of the arm.

The purpose of this study was to identify the neuronal representation of texture in the barrel cortex of actively behaving rats. Experiments were performed in an arena illuminated only by infrared light, thereby eliminating potential visual cues. To discriminate textures, rats perched at the edge of an elevated platform, extending their whiskers across a gap to touch a textured plate mounted on a second platform. Gap length, around 15 cm, was great enough that on nearly every trial, they could reach the textured surface only with the long whiskers of the snout—the macrovibrissae. Rats were trained to execute different actions according to the texture they contacted—smooth or rough. In the “1-arm task” (Figure 1A), rats had to withdraw and turn to a water spout. The texture identity indicated whether a left or right turn was correct. In the “3-arm task” (Figure 1B), they had to either cross to the opposite platform (if the contacted texture signaled the presence of the reward on that platform), or withdraw and proceed to a second gap at which the reward texture was present. In this task, we examined behavioral and neuronal data only from the first gap encountered on each trial. In both tasks, rats received a water reward when they performed the correct action. Each rat was trained only in one task (1-arm or 3-arm), and the learned association was fixed over time for each rat (e.g., in the 1-arm task, rough—turn left, smooth—turn right).

**Figure 2:**
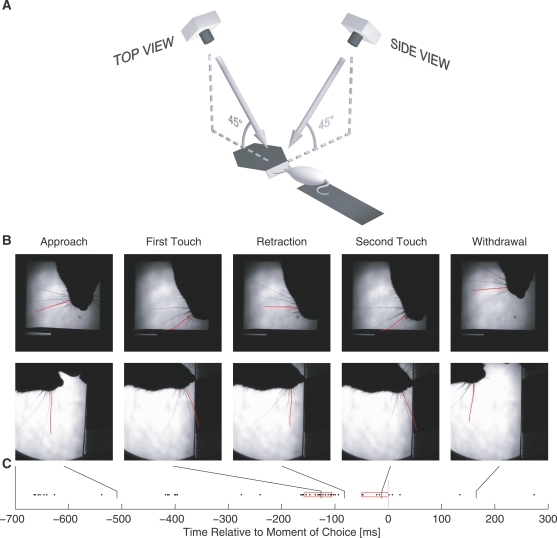
Behavior and Neuronal Activity in a Typical Trial (A) Spatial arrangement of the two high-speed cameras. (B) Images from high-speed films taken from two different views (top row—top view, bottom row—side view, see [A] for positions; frame pairs of the upper and lower rows are synchronous). Whisker C2 is traced. Although in the side view, the rat's snout seemed to touch the texture, the corresponding top view frames show that this was not the case. Although on a small proportion of trials, rats were close enough that microvibrissae contact could not be excluded, this happened too rarely to explain the animals' high performance. (C) Spikes fired by a neuronal cluster whose receptive field was centered on whisker C2. Estimated moment of choice is given by gray vertical line at 0 ms. Contacts of the selected whisker before decision are marked as red frames. Note the increase in firing rate during contact. Contacts after choice are not highlighted, and the corresponding spikes were not considered in the analysis. Shown: rat 16, session 2006-07-23, cluster #58, trial 20, smooth texture.

One typical trial in the 1-arm task is illustrated in Figure 2. To constrain the rat to use the whiskers providing input to the recorded cortical neurons, we had clipped most whiskers on both sides of the snout except for the C row (see Materials and Methods for details). We monitored rat behavior by filming the head and whiskers [10] with a pair of high-speed cameras (1,000 frames/s) as the rat probed the texture (Figure 2A). Top- and side-view images show crucial behavioral events (Figure 2B; complete films can be viewed in Videos S1, S2, and S3). At the outset, the rat approached the discriminandum (first frame), then it touched the plate with whisker C2 (second frame). Next, the whiskers retracted while the snout remained in the forward position (third frame). Whisker C2 made a second touch (fourth frame), and finally the rat withdrew to collect a water reward from a drinking spout (last frame).

During the session illustrated above, we recorded the activity of a neuronal cluster from barrel cortex (electrode depth: 700 μm; principal whisker: C2). Below the film images, the spike train on the corresponding trial is illustrated (Figure 2C). The instant when the rat made the first detectable movement signaling identification of the texture (i.e., snout withdrawal) was set to 0 ms and aligned to the gray vertical line. This is referred to as the moment of choice. Times of the film frames are projected down to the spike train, and time windows of whisker C2 touch are highlighted by red frames.

**Figure 3:**
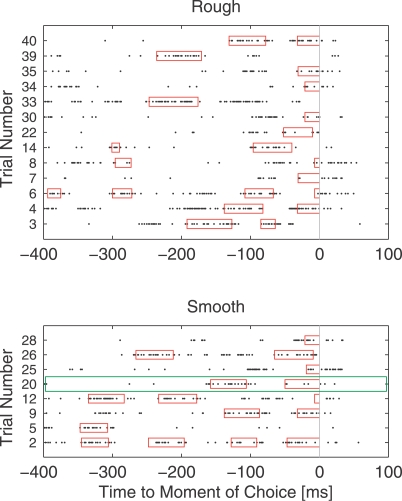
Activity of the Same Neuronal Cluster in the Course of a Session Trial 20, depicted in Figure 2, is highlighted by a green box. Spike times are relative to moment of choice (gray line). Contacts of whisker C2 occurring before choice are marked by red frames. Note the increase in firing rate during contact. On missing trial numbers, whisker contact times could not be defined.

Spike trains collected from the same neuronal cluster in all trials of this session with discernible touches—13 rough trials (upper panel) and eight smooth trials (lower panel)—are shown in Figure 3. Trial 20 (green box) was illustrated in Figure 2. The moment of choice on every trial was set to 0 ms and aligned to the gray vertical line. Whisker C2 touch times are highlighted by red frames, and it is evident that firing rate increased when the whisker touched the plate.

### Whisker Contact with Textures

**Figure 4:**
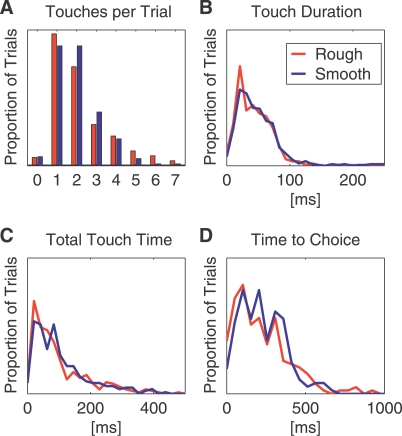
Whisker Touch Statistics For the whiskers of interest, contact onset and offset times were measured for all touches ending prior to the moment of choice. (A) Number of touches per trial per whisker (red—rough texture; blue—smooth texture) (B) Individual touch durations. (C) Summated duration of all touches of a single whisker per trial. (D) Time from first contact of the whisker of interest to the rat's choice. Values are consistent with a reported range of 150 to 500 ms in a different texture discrimination task [34].

We analyzed the high-speed films to extract the times of whisker contact with the plate. For each session, we monitored those whiskers that were used by the rat to extract texture and that were in the receptive field of responsive neuronal clusters. The full dataset of whisker touches comes from two rats (rat 1, 3-arm task; and rat 16, 1-arm task) and consists of 20 sessions containing 701 trials, 402 of which had touches with clear onset and offset; the total number of touches was 1,210, from whiskers C1, C2, D2, and D3, where the caudal whiskers of either the C or the D row were present in a given session. Figure 4 summarizes the findings: On a typical trial, the rat made 1–3 touches of 24–62-ms duration each before making its choice, summating to a total touch time per whisker of 88–224 ms; the time from first whisker contact to the choice action was 98–330 ms (interquartile ranges). We then asked whether any of these parameters differed according to the texture presented to the animals. Statistical tests (Mann-Whitney-Wilcoxon tests, *p* > 0.05) showed that rats exhibited the same distributions of contact parameters for rough and smooth, suggesting that motor output was not modulated by the encountered texture.

### Texture Coding during Whisker Contact

During the texture discrimination sessions described above, we obtained 54 neuronal recordings from barrel cortex (five of them judged as single neurons according to conventional measures and the remainder multiunit clusters). There were, on average, 23 analyzed trials per cluster.

To find out how firing rate was modulated by contact with the textured plate, we first define a cluster's contact index *C* as the touch-evoked firing rate, averaged across rough and smooth trials, normalized to the whole session's mean firing rate:

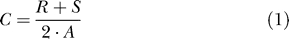
where *R* and *S* are the mean firing rates across all rough and smooth touches, respectively, and *A* is the session's average firing rate (including intertrial periods). During contact, spikes were counted from 4 ms after touch onset to 4 ms after touch offset, in line with the response latency we observed in later analyses (see below). For the neuronal cluster of Figure 3, *C* = 8.1.

Further, we define a cluster's texture index *T* as the firing rate difference between rough and smooth trials normalized to the sum of those two rates:

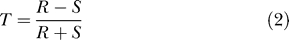



A value of *T* between −1 and 0 signifies a higher firing rate during smooth touches, whereas a value between 0 and 1 signifies a higher firing rate during rough touches. For the same neuronal cluster, spiking density was higher during contacts with the rough texture than with smooth, reflected by a value of *T* = 0.050.

**Figure 5:**
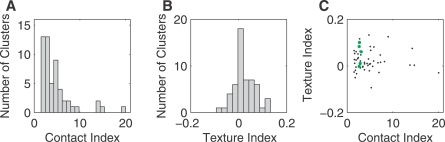
Contact and Texture Indices on Whisker Touch (A) Distribution of the contact index, *C*. All clusters have *C* > 1, equivalent to an increase in firing rate on contact. (B) Distribution of texture index, *T*. Most clusters have *T* > 0, equivalent to a higher firing rate for rough than for smooth contact. (C) Correlation between contact index and texture index across clusters. The green points are single neurons.

The distributions of *C* and *T* indices for all clusters are shown in Figure 5A and 5B, respectively. The fact that all *C* values are greater than one indicates that neuronal clusters encoded contact with the texture plate, whether rough or smooth, by an increase in firing rate. If there were single neurons inhibited by contact, the suppression may have been disguised by the activity of other units in the multineuron cluster. However, among the studied single neurons, none was inhibited by contact. The mean value of *C* was 3.81. Contact coding could serve many functions, such as providing a signal to support object localization [5,11,12].

Beyond this, the neuronal clusters encoded the identity of the plate by a differential firing rate for rough and smooth. The majority of clusters produced a higher firing rate for rough than for smooth contacts (*T* distribution skewed to positive values). The mean value of *T* was 0.051, a value significantly different from what would be expected if texture had no influence on firing rate (*p* < 0.005, permutation test). This value of *T* corresponds to a mean difference between rough and smooth firing rates of 10%.

Considering only the single neurons among these data, we found similar texture coding (*T* = 0.085, significantly greater than chance, *p* = 0.008). Whereas the single-unit session-average firing rates spanned a wide range (4.6–43 spikes/s), they all reacted to whisker contact with a robust increase in firing rate: their contact indices *C* all lay between 2.6 and 3.1 (see green points in Figure 5C).

The present finding that neurons encode surface coarseness by firing rate, and that the rate is higher for rough rather than smooth textures, confirms results from studies using artificial whisking on textures in anesthetized rats [8,9].

The correlation between *C* and *T* indices within these 54 clusters (Figure 5C) was close to zero and insignificant.

#### Response dynamics.

**Figure 6:**
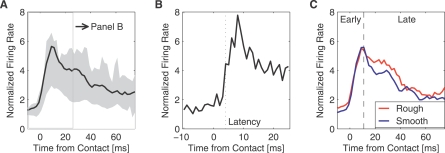
Dynamics of Neuronal Response During Whisker Contact Spike times were aligned to the onset of each whisker contact; onset time was set to 0 ms. Touches had a wide range of durations (see Figures 3 and 4) so that response values at progressively later times were gathered from progressively fewer contacts; only 30% lasted longer than 80 ms. (A) Quartile plot of firing rates. The interquartile range (25% to 75% of neuronal clusters) is shaded gray; the black line is the mean. All clusters were normalized by dividing by their average rates across the whole session. (B) Magnified view of mean response onset. (C) Response dynamics separated by texture.

To explore the temporal evolution of signals in the behaving rat, we built a peri-contact time histogram (PCTH) by aligning spikes to the instant of whisker contact with the plate, judged from high-speed films (Figure 6A). In order to show all neuronal clusters together, firing rates were normalized by dividing by each cluster's whole-session average rate. The fact that firing rate just prior to contact rested at the normalized value of one indicates that neurons assumed baseline values of firing rate in the interval before and between whisker contacts. Responses began 4 ms after onset of contact (Figure 6B; the firing rate in the bin at 4 ms was higher than all 1-ms bins from −50 to 0 ms), which, in the limits of the resolution of our analyses (1 ms), is consistent with a value of 5 ms for layer 4 neurons reported in anesthetized rats [13]. This indicates that response onset dynamics in layer 4 are similar in anaesthetized and awake animals. Responses showed a marked onset peak followed by a gradual falloff after about 11 ms.

With what time course did the texture-specific firing-rate difference develop? To answer this, we plotted touch-evoked activity in two separate traces corresponding to rough and smooth touches (Figure 6C). This revealed no texture-related difference during the initial, sharply rising response phase (4–11 ms, marked “early” in Figure 6C: *T* = −0.006, *p* = 0.6, permutation test). Shortly thereafter, a greater firing rate for rough touches (red trace) compared to smooth touches (blue trace) became evident (from 11 ms to the end, marked “late”: *T* = 0.061, *p* = 0.003). Integrated across the whole touch (from 4 ms to the end), the firing-rate difference between rough and smooth was significant (*T* = 0.051, *p* = 0.007; permutation tests), as shown previously in Figure 5B.

### Texture Coding Preceding Choice

In the previous section, neuronal activity precisely aligned to contact times revealed a higher firing rate during rough touches than smooth. In the next analysis, we suppose that the rats integrated spikes without exact knowledge of specific touch times. We ask whether the posited firing rate code could support discrimination between the two textures even in the absence of touch-time knowledge. This choice-triggered analysis aligns trials at the moment of choice and explores any texture-specific signals present in neuronal firing rate in the preceding interval.

**Figure 7:**
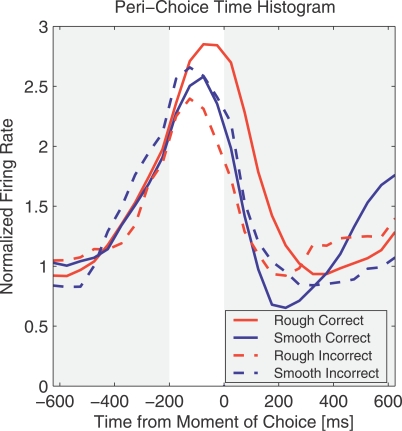
Average Normalized Firing Rate as a Function of Texture Firing rates were aligned to the moment of choice (0 ms) and binned in a sliding 250-ms window, centered on each data point. Each cluster's firing rates were divided by the recording session mean rate (note the value of 1.0 before whisker contact, at −600 ms), then the average was calculated across all clusters. The unmasked area, from −200 to 0 ms, shows the strongest texture coding before the rat's choice. Texture-related differences after the rat's choice were a consequence of the different actions rats performed in correspondence with each texture (e.g., turn left or turn right) and were not further analyzed.

The analysis included three animals—rats 1 and 16 from above, plus a third animal, rat 10. The latter had all its whiskers intact, which made the detection of individual whisker touches impractical. Therefore, it was excluded from contact-time analysis but was suitable for choice-triggered analysis. For these three rats, based upon 77 neuronal clusters (eight of them single neurons) and 868 trials, Figure 7 shows the average normalized firing rate around the moment of choice. On correct trials (solid lines), the firing rates on rough and smooth trials diverged about 200 ms before the moment of choice, with a higher firing rate on rough trials.

The presence of a texture signal in barrel cortex, averaged across correct trials, leaves open the question of whether the rats used the signal to guide their behavior. One strategy to address this question is to ask whether the variance in sensory signals was connected in any way to the decision expressed by the animal. Did the representation of texture differ on incorrect trials? The dashed lines in Figure 7 show that the relation of firing rate to texture reversed on incorrect trials: smooth greater than rough.

**Figure 8:**
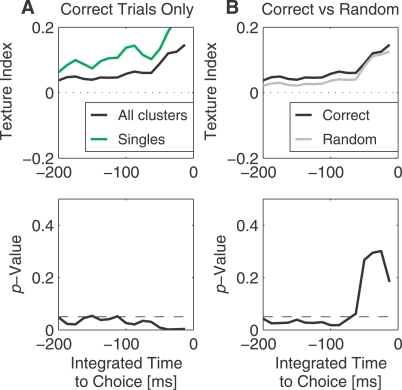
Texture Coding Before Choice (A) Upper panel: texture index *T* calculated for backwards-growing windows from 0 ms (moment of choice) to −*t*, averaged across correct trials only; Black trace: average across all clusters; green trace: single neurons only; Lower panel: significance of *T* > 0 for all clusters. The dashed line shows the 5% limit. (B) Texture index *T* on correct trials only (black trace, carried over from [A]) versus random trials (gray trace); Lower panel: significance of the difference between correct and random trials.

In order to quantify the difference in response to the two discriminanda, we calculated a texture index *T* analogous to the one used above for the touch-based analysis:

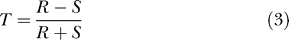
where *R* and *S* are the average firing rates for rough and smooth trials, respectively, in a window reaching from 0 ms (choice time) back to *t* ms prior to moment of choice. Figure 8A, upper panel, shows the average texture index, on correct trials, as a function of the time window *t*, averaged across multiunit clusters and single units. The index is significantly greater (*p* < 0.05 for most time windows) than what would be expected if texture had no influence on firing rate (Figure 8A, lower panel).

For time windows around −75 *ms*, *T* ≈ 0.06, a value close to the touch-delimited texture index (0.051; see “Texture Coding during Whisker Contact”). As few contacts are longer than 75 ms (see Figure 4), the 75-ms time window would be expected to approximately capture the last touch before the animal's choice.

For *T* in Figure 8A, we considered only correct trials, but we then examined whether neuronal coding differed in incorrect trials. Figure 7 suggests a difference between correct and incorrect trials, but to statistically show its significance, it was inappropriate to compare their values directly; the set of incorrect trials in a given session sometimes included only one stimulus category, rough or smooth, making it impossible to calculate any statistic based on their difference. Therefore, we chose to first calculate *T* for correct trials only and compare it to the index calculated on a random subset of trials containing a mix of correct and incorrect trials; the fully correct and random subsets contained the same number of trials. Figure 8B, upper panel, shows that the texture index *T* on correct trials was higher than on random trials, meaning that on incorrect trials, the neuronal clusters carried a degraded texture signal. The difference was significant in time windows equal to or longer than 75 ms (Figure 8B, lower panel; permutation test).

#### Texture Coding in Population Measures.

Rat 10, as mentioned above, had all whiskers intact. This allowed the rat to use a different set of whiskers from trial to trial. Responses in a single barrel would be expected to transmit an inconsistent signal because on a given trial, the principal whisker of the neuronal cluster of interest might or might not palpate the surface. In other words, whereas the texture signal in whisker-trimmed rats would be “channeled” through the same small set of cortical barrels on each trial, in rat 10, the signal would be dispersed across a large and varying set of barrels. Consistent with this reasoning, the texture index *T* was not significant when rat 10 was considered alone (unpublished data). However, the above interpretation suggested that a measure of broader population activity might uncover a clearer texture-related signal. For this reason, we recorded a local field potential (LFP) from barrel C2/3 at a depth of 700 to 850 μm in this rat, thereby sampling the activity of many neurons across a wide territory. Previous studies [14] have estimated the diameter of the catchment volume to be around 1 mm, which could sample from many of the barrels surrounding the recording site.

**Figure 9:**
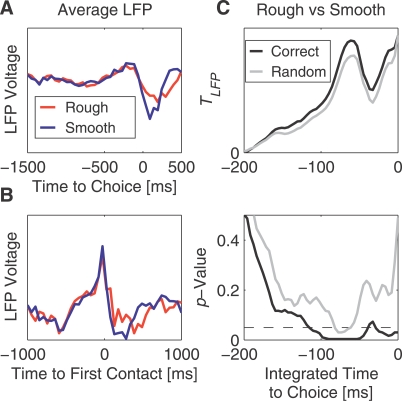
Texture Coding in Local Field Potential (LFP) (A) LFP curves aligned on moment of choice and averaged across trials. (B) LFP curves aligned on moment of first touch of any whisker. (C) Upper panel: LFP Texture Index *T_LFP_* on correct trials only compared to random trials, integrated across backwards-growing windows from the moment of choice to −*t*. Lower panel: significance of *T_LFP_* being greater than chance on correct trials (black trace) and significance of *T_LFP_* being greater on correct trials compared to random trials (gray trace). For both, *p* < 0.05 for *t* = −75 *ms*.

Figure 9A shows LFP curves from six sessions of rat 10, with a total of 493 trials. The voltage-traces were resampled at 20 Hz, averaged across trials, and aligned to the moment of choice. The sharp peak around the moment of choice indicates that the LFP signal represented activity relevant to the task for either texture. Similarly, LFP traces aligned on first contact (of any whisker) showed a clear contact response (Figure 9B; data from one session only, 97 trials) consistent with previous reports [15].

Figure 9A suggests that, for a time window of 100 ms before choice, the rough trace was above the smooth trace.

To quantify this difference, we defined an LFP texture index *T_LFP_* similar to the spiking texture index *T* used above:

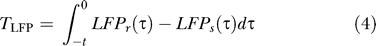
where *LFP_r_* and *LFP_s_* are the average LFP traces, resampled at 250 Hz, on rough and smooth trials, and *t* = 0 the moment of choice.

In Figure 9C, top panel, the black trace shows *T_LFP_* for correct trials. It is greater than zero and significantly greater than expected by chance for time windows from 0 to around −75 ms (Figure 9C, bottom panel). Thus, the LFP traces differed significantly between rough and smooth trials.

The gray trace in Figure 9C, top panel, shows *T_LFP_* for a random subset of trials, including incorrect ones. *T_LFP_* was lower on random trials than on correct trials, and the difference becomes significant for time windows from choice to around −75 ms.

#### Texture information.

Averaging across trials allowed us to identify firing rate as a clear correlate of the texture discriminanda on correct trials and to show that on trials when the rat made an error, firing rate deviated from the cardinal “correct” values. Average LFP findings in rat 10 were analogous. The rat, however, cannot average firing rates across trials, but must make an immediate decision using the signals generated on each single trial. In this final section, we examine single-trial signals.

Shannon's Mutual Information [16,17], hereafter referred to simply as information, can be used to measure how much a neural response reports about a stimulus, on a single trial [18].

Because rats showed a clear difference in firing rate between rough and smooth trials in the analysis of Figure 8, we applied information analysis to spike rates integrated across the same backwards-growing windows. Information was calculated for each cluster individually, classifying the spike count on each trial into one of two categories: greater or less than median.

**Figure 10:**
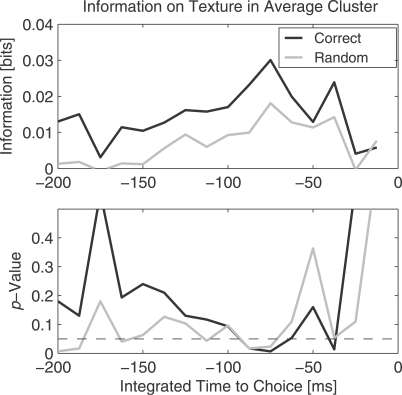
Texture Information Carried by Firing Rate Upper panel: information about the two stimuli transmitted by firing rate, averaged across clusters, using backwards-growing windows as in Figure 8A, for correct only and random trials. Lower panel: significance of the information on correct trials being greater than chance (black trace) and significance of the information on correct trials being greater than on random trials (gray trace); *p* < 0.05 for time windows from −75 to 0 ms. The dashed line shows the 5% limit.

In Figure 10A, upper panel, the black trace shows the information carried about the stimulus on correct trials, averaged across all neuronal clusters. The information was significantly greater than chance (*p* < 0.05 around −75 ms, see lower panel; permutation test). The gray trace shows the information on random trials, which was consistently lower, and the correct-random difference was significant around −75 ms (permutation test).

The peak amount of information was reached for a time window from −75 to 0 ms, where 0 ms was the observed moment of choice. In this window, the average multiunit cluster's spike count carried 0.03 bits of information about the stimuli—3% of the information the rat would require in order to perfectly identify the stimulus (two stimuli correspond to 1-bit entropy). When calculated using all trials, including those with incorrect rat choices, the maximum information was lower, 0.021 bits (significantly greater than chance, *p* < 0.05, permutation test). However, the information about the stimulus extracted by the rat, estimated by the mutual information between the stimulus and the rat's choice, was 0.46 bits (88% correct trials). Thus, the information carried by the average neuronal cluster was about 0.021/0.46 (≈4.6%) of the information actually used by the rat.

To summarize, neuronal clusters carried significant single-trial texture information by firing rate, and this information was degraded when incorrect trials were included in the analysis, in line with the collapse of the texture index on random trials (Figure 8). This suggests that the recorded neuronal signals were part of the flow of information leading to the rat's decision.

### Origin of Degraded Signal on Incorrect Trials

In all choice-triggered analyses, there was a significant correct–incorrect difference, as revealed by the correct versus random statistics. The spiking texture index *T*, Shannon's information calculated on these same spikes, and the LFP, all showed a degradation of the rough/smooth distinction on incorrect trials (Figures 8, 9, and 10).

In all the measures of touch-evoked spiking reported in Figures 5 and 6, no significant difference existed between correct and incorrect trials. In other words, on incorrect trials, whisker touch elicited the same rough and smooth firing rates as on correct trials (unpublished data). However, examination of firing rates aligned to the animal's choice, rather than to touch times, showed an inversion of the texture-related firing-rate difference on incorrect trials, with more spikes on smooth trials than on rough trials (Figure 8). As the touch-evoked firing rate was not altered on incorrect trials, what caused the inversion of choice-aligned firing rates?

We hypothesized that a change in contact duration and/or number of whisker contacts was the determining factor. To test this, we computed the contact fraction *f*, defined as the cumulative duration of all whisker contacts during a 75-ms prechoice window, divided by the duration of that window. A value of zero means no touch, and *n* whiskers touching for a fraction *x* of the whole duration yield a value of *nx*. Table 1 shows average values for rough, smooth, correct, and incorrect trials.

**Table 1:**
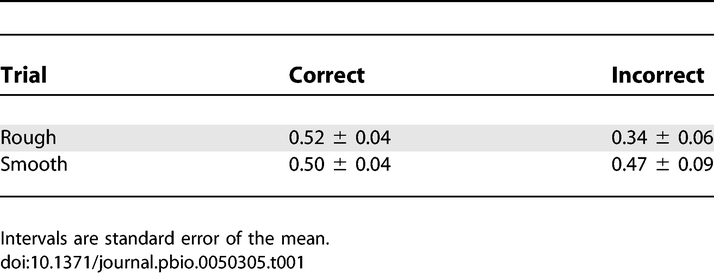
Whisker Contact Fraction *f*

Although for smooth trials, there was no significant difference between correct and incorrect trials (*p* = 0.46), for rough trials, the touch fraction was significantly reduced on incorrect trials (*p* < 0.05, permutation test). This matches the findings of Figure 7, where on rough trials, the firing rate was substantially lower on incorrect trials compared to correct trials, whereas on smooth trials, there was no striking difference. Thus, it seems, when the rats mistook a rough texture for a smooth one, they did so by “decoding” a mistakenly low firing rate in barrel cortex, which in turn was a consequence of missed whisker contacts.

## Discussion

### Neuronal Signals Representing Texture

We trained rats to make texture discriminations with their long facial vibrissae and investigated how barrel cortex neuronal clusters carried the sensory signal. Rats perched at the edge of an elevated platform to project their whiskers across a gap and touch a textured surface mounted on a second platform [4,19]. To receive a reward, they had to make a choice dictated by the identity of the texture, rough or smooth.

On a typical trial, the rat made 1–3 touches of 24–62-ms duration each (interquartile ranges) before making its choice. The first analysis was based on barrel cortex activity aligned to these precise whisker contact times. Neuronal clusters showed a sharp rise in firing rate whenever a whisker in their receptive field contacted the textured plate, with a latency of about 4 ms. Contact responses are consistent with previous studies reporting responses to active touch in terms of spikes [15,20] and membrane potential [12,21]. Whereas the early contact response showed no sensitivity to texture, a texture-specific difference in firing rate emerged around 11 ms after response onset, with a higher rate for rough textures.

Thus, firing rates during contact discriminated the two textures; but rats may not have precise knowledge of whisker contact times. Is firing rate a plausible code for texture in the absence of such knowledge? To answer this, our second analysis determined whether firing rate in the period leading up to the rat's observed choice distinguished the two textures even with touch times unmarked. In practice, we measured firing rate in a window growing backwards in time from the moment of choice for up to 200 ms. Across this window—and especially during the 75-ms window preceding the animal's choice—firing rates were higher on rough than on smooth trials, confirming this as a valid coding mechanism whether or not the rat has access to exact touch-time information.

In this choice-triggered analysis, we quantified the texture information transmitted by firing rate using Shannon's Mutual Information [16,17]. The average neuronal cluster carried 0.03 bits, 3% of the information (1 bit) necessary to provide full knowledge of stimulus identity. In relation to the knowledge of stimulus identity expressed by the rats' behavior (0.46 bits), the average neuronal cluster carried 4.6% of information.

From results obtained in anesthetized rats [8,9], we expected that roughness would be encoded by firing rate in the awake, behaving animal as well; roughness coding by firing rate has also been suggested in primates [22–24]. In particular, we predicted a higher firing rate during whisker contact with the rough surface. The current results match that prediction and show that the information carried by firing rates is significant. Additional information may be available from spike patterns and correlations among neurons [9,25–27], and this supplementary coding mechanism could be particularly important in supporting discrimination between textures that are more similar in coarseness than the rough versus smooth stimulus pair used here [4].

The measured texture-related response difference in multiunit activity was more reliable in rats that had all but a few whiskers trimmed (rats 1 and 16). On the other hand, in a rat whose full whisker array was left intact, the texture difference was highly significant in the LFP (rat 10). In other sensory systems, the cortical LFP has been found to reflect activity distributed across a spatially wider territory as compared to spiking activity. For instance, in cat auditory cortex, receptive fields measured by LFP signals were twice as wide as those measured by spiking activity [28]; similarly, in monkey striate cortex, the spatial decay constant of an LFP was 0.5 mm [14], compared to 0.05 mm for multiunit spiking activity [29]. The difference in spatial sampling could explain the apparent discrepancy between LFP and neuronal clusters in our data. Possessing many vibrissae, rat 10 touched the texture with combinations of whiskers that, on many trials, did not include the whisker projecting to the barrel in which the electrode was positioned. Whereas the neuronal cluster activity on such trials would not contain any texture signals, the LFP could pick up activity from neighboring barrels whose whiskers did touch.

For the LFP as well as for the multiunit spike data, the most reliable texture-related signal was found for a time window of only 75 ms preceding choice; this window length would typically contain the last whisker contact before the execution of the choice. As time windows reached progressively farther back, the trial-to-trial variability in the number of touches may have acted as noise, masking the texture-related difference. An alternative interpretation is that the large amount of information contained in this final interval is exactly what led the rats to make their choice.

### From Whiskers to Cortex

A complete understanding of texture coding will require a detailed analysis of how the sense organ interacts with different surfaces. In earlier work, we noted that movement of a whisker across a given texture gave rise to a vibration at the whisker base with a “kinetic signature” characteristic of the contacted surface [8,30]. Textures of similar coarseness induced kinetic signatures distinct from each other by the temporal pattern of high-velocity events, whereas textures of differing coarseness, like those utilized here, induced kinetic signatures distinguished by total energy. In the trigeminal ganglion and barrel cortex, the temporal pattern of kinetic events was translated into a temporally aligned pattern of spikes, whereas the energy of whisker vibration was translated into the neuronal firing rate [9]. The power spectrum of multifiber discharge from the vibrissal sensory nerve in anesthetized rats also shows texture-specific features [31]. By temporal correlation techniques, we also determined that texture signals were relayed rapidly from subcortical structures to barrel cortex, as opposed to emerging more slowly from within intracortical circuits (Figure 3C of [9]).

Whisker resonance has been proposed as a texture discrimination mechanism; the amplitude of oscillation could vary according to the relation between a texture's spatial features and the whisker mechanical properties [32]. Our analysis of whisker movement focused on the onset and offset of contact with the texture and did not attempt to measure whisker vibration. Thus, our data neither confirm nor refute the resonance hypothesis. However, during examination of high-speed films, we did not observe vibrations suggesting whisker resonance. Identifying exactly which kinetic features cause a texture-specific difference in firing rate is a question of current interest.

Barrel cortex is critical to texture discrimination. After rats were trained to discriminate between sandpapers with grain sizes of 0.4 mm in diameter (200 grains/cm^2^) and 2 mm in diameter (25 grains/cm^2^), their performance was abolished by a lesion of barrel cortex [33]. The deficit was selective to whisker capacities, since the rats with lesions adopted a strategy of using their forepaw to touch and identify the textures. Although barrel cortex is known to be an essential processing step in texture judgment, the underlying neuronal representation has been difficult to uncover in behaving rats. When rats were trained to discriminate between a smooth surface and one containing grooves of width, depth, and spacing of 250 μm, barrel cortical activity recorded during texture contact showed no robust correlate of texture, measured either by overall firing rates or temporal discharge patterns [34]. This negative finding can be reconciled with our results by noting that the rat described in that study had all whiskers intact (like our “LFP rat”), and that precise whisker contact information was not available due to the low time resolution of the video analysis (60 frames/s as opposed to 1,000 frames/s in the present experiments).

### Active Tactile Sensation

It seems clear from our results that the quantity of texture information carried by neuronal cluster firing rate in the behaving animal is smaller than that in anesthetized animals [8,9]. In anesthetized rats, cortical clusters on average carried, for smooth surface versus P100 sandpaper (which may be likened to the smooth and rough discriminanda used in the present experiments; however, it should be noted that the rough surface in the present experiments was a mold of P100 and was not as coarse as the surface from which it was pressed), about 0.25 bits of information per 125-ms whisk (Figure 8 in [9]). This is eight times greater than the 0.03 bits, on average, contained in the spike count in the final 75 ms prior to the moment of choice measured in the present work (Figure 10). This discrepancy can be explained by the fundamentally different experimental conditions. In the anesthetized rat, the animal's head was immobile, the textures were always positioned at a fixed distance from the base of the whiskers, and there was little whisk-to-whisk variability—movement was evoked by controlled electrical stimulation of the facial nerve. Within one category of texture, there was only minimal trial-to-trial stimulus variability. Low stimulus variability led to correspondingly low response variability which, in turn, increased the rate of information transmission. In contrast, in the behaving rat, the angle of approach and the distance from snout to texture varied from trial to trial. Moreover, the amplitude, frequency, and speed of whisker motion were all under the rat's own control. All of this caused higher response variability and reduced texture information.

However, the variability that appears as noise to the experimenter may be interpretable to the animal if it can access information about its distance from the plate and its self-generated whisking motion. Evidence has been accumulating both for a distance signal [35] and for a whisking signal in barrel cortex [2,12,36–38]. The distance signal is relayed by the sensory afferents. The whisking signal, in contrast, could originate either as an efferent copy relayed from the motor system to the sensory system [39] or as a sensory signal from the whisker follicle [40]. In summary, the rat's knowledge of spatial coordinates and of its own motor output could make the sensory input more reliably decodable.

Knowledge of motor output could be used as follows. Intentionally or not, the rat may generate “motor noise” by whisking with more or less force from trial to trial. Stronger whisks are likely to provoke more afferent spikes, but if barrel cortex or a later processing station collects information about the strength of each whisk, it can recalibrate the sensory input and avoid errors (e.g., mistaking the smooth texture for rough on trials when strong whisks cause a firing rate greater than the smooth average). This mechanism may reconcile the rat's high behavioral performance with the low single-trial reliability of the observed sensory signal.

### Information in This and Other Discrimination Tasks

Many studies have measured mutual information between neuronal firing rates and stimuli; here, we considered only those in which, like in our experiments, the animal was performing a discrimination task.

When monkeys discriminated the frequency of flutter-vibration stimuli applied to the fingertips [41], cortical neurons carried in their firing rates 0.28 bits (in primary somatosensory cortex [SI]) and 0.14 bits (in secondary somatosensory cortex, [SII]) of information about the frequency, on average. There were eight frequencies, so this information corresponds to 9.3% (SI) and 4.6% (SII) of the stimulus entropy (log_2_8 = 3 bits). Information carried by SI neurons was significantly lower when the animal was not doing the discrimination task but merely passively attending the stimulus.

In an olfactory discrimination task in monkeys [42], neurons in orbitofrontal cortex carried, in their firing rates, 0.06 bits of information on average about the identity of eight odors, equivalent to 2% of available information.

In monkeys performing a visual discrimination task with five different objects [43], neurons in the inferior temporal gyrus carried, on average, 0.26 bits in their firing rate, equivalent to 11.2% of the stimulus entropy.

Neurons in the monkey lateral intraparietal area (LIA) [44] carried 0.1 bits (10% of the entropy) about the location of a sound stimulus and 0.3 bits (10% of the entropy) about the location of a visual cue.

Thus, across the various cortical areas probed, neurons carried in their firing rate between 2% and 11% of the available stimulus entropy. The barrel cortex neurons in our recordings carried 2.1% of the available stimulus entropy (3% on correct trials only), which is within the range of information values reported in a wide range of cortical sensory areas. The fact that, in most of the experiments cited above, the animals passively received experimenter-controlled stimuli—as opposed to stimuli generated by our actively whisking rats—could account for the moderately higher information rates.

### Behavioral Relevance of Texture Signals

In spite of extensive training, in some rats, performance did not exceed 80%. An incorrect choice on the remaining trials could occur because the neurons in barrel cortex held a poor stimulus representation on those trials; alternatively, barrel cortex could generate an equally accurate representation on each trial, but this representation could be inaccurately “read out” by the downstream brain systems that generate a behavioral response. A comparison of the signals (firing rate and LFP) carried in the correct and incorrect trials helped to distinguish between the two alternatives. On correct trials, average firing rate immediately before choice was higher for rough than for smooth; on incorrect trials, this was reversed. As a consequence, the texture information carried by neuronal firing in correct trials was greater than that of a random subset of trials, showing that incorrect trials occurred at least partially due to low stimulus signal quality. In other words, when barrel cortex neurons carried the “wrong” signal, the rat was likely to make a wrong choice, and when they carried the “correct” signal, the rat was likely to make a correct choice.

What, then, was the origin of the inappropriate stimulus representation on incorrect trials? The cause could be an errant afferent signal: the rat may have palpated the texture more or less forcefully than intended or, by positioning itself incorrectly, might have missed it altogether. Alternatively, central modulatory inputs could cause stimulus-independent fluctuations in barrel cortex firing rate. Although our data do not allow evaluation of the latter scenario, the analysis of touch times in the last part of Results, “Origin of Degraded Signal on Incorrect Trials,” suggested that missed touches are one of the causes of incorrect trials. Although the texture signal *T* during successful whisker contacts did not differ between correct and incorrect trials, the amount of whisker contact did: on rough incorrect trials, the whiskers were on the texture for significantly less time during the last 75 ms before choice than on correct trials. As rats whisked continuously, the non-touches must have been missed attempts to contact the plate due to inappropriate posture or positioning. Instead of recalibrating its choice according to knowledge of the missed contact, the animal may have interpreted the low firing rate as an indication of a smooth texture.

Even though the same reasoning cannot apply to smooth incorrect trials, these results strongly suggest that the rat was not able to decode firing rate according to knowledge of individual contacts: some amount of variability in afferent signals (successful or unsuccessful touches) was beyond the control of the animal and therefore acted as a source of behavioral error.

Additional study is required to understand how whisker dynamics evoke cortical spikes and why spiking signals vary across trials. However, our data strongly suggest that, in the present texture discrimination task, barrel cortex firing rate is directly involved in the process that leads from sensation to behavior.

## Materials and Methods

### Subjects.

Wistar rats (Harlan Italy, S. Pietro al Natisone, Italy) weighing about 300 g were housed individually and maintained on a 13/11-h light/dark cycle. Food was restricted to 15 g of rat chow (Harlan) per day; throughout the experiment, rats continued to gain weight. Water was given during training as a reward and was also available ad lib for 1 h after training. All experiments were conducted in accordance with National Institutes of Health, international, and institutional standards for the care and use of animals in research and were supervised by a consulting veterinarian.

### Apparatus.

The arena was situated in a Faraday cage and was illuminated by light-emitting diodes (LEDs) emitting infrared light (wavelength >880 nm) in which albino rats have negligible visual function. The apparatus was constructed in aluminum and consisted of three rectangular platforms (36 × 11 cm) distributed radially about a central hexagonal platform (side length, 10 cm). Each rectangle's shorter edge faced the hexagon across a gap of adjustable width. Platforms were elevated 30 cm above a table.

Discriminanda were fabricated in two ways.

For the 3-arm task, a 3 × 10 cm sheet of P100 sandpaper (mean grain size, 162 μm) was glued onto an acrylic glass plate. This was used to form a mold to cast true copies of the sandpaper texture with a bicomponent resin. This yielded a 0.3 × 3 × 10 cm plate of resin, one side of which had a P100-like surface. To build a smooth discriminandum, the mold was made of the acrylic glass plate without sandpaper. These discriminanda were mounted vertically.

For the 1-arm task, P100 sandpaper was pressed onto a heated 3 × 10 cm acrylic glass plate. This procedure left each plate with a smooth side and a rough side (a negative mold of P100). These discriminanda were mounted at a 45° angle with the vertical, for some sessions facing up, for some facing down.

Both methods yielded pairs of objects of the same size, overall shape, and odor. The performance of subjects was equivalent for discrimination of both types of discriminanda and all mounting angles.

### Texture discrimination task.

For one week, each rat was handled and habituated to the training arena under dim visible light. Then, the visible light was switched off and only invisible infrared illumination remained. For the rest of the experiment, training sessions were held once a day for about 1 h, usually during the dark phase of the light/dark cycle. The goal of training was for the rat to learn to discriminate between the smooth and rough textures using the long whiskers of its snout.

In some rats, most of the whiskers were clipped. This was done to encourage them to use the whiskers that were in the receptive field of recorded neuronal clusters. Whenever this was done, all long whiskers (Greek and arcs 1–4; not arcs 5 and beyond, which are difficult to see and cut in an awake rat) on both sides were clipped, except for the row of interest, which was C or D.

Potential olfactory cues were removed from textures by washing them at least once every session. Within and across sessions, different plates from within one texture category were exchanged to ensure that rats discriminated by the category of texture rather than by cues (tactile or non-tactile) linked to individual objects.

*The 3-arm task.* The rat started a trial in one arm of the 3-arm maze. It had to jump to the central platform (Figure 1B (i)) crossing the gap of 12–15 cm. From the central platform, the rat could choose one of the two remaining arms by crossing another gap. On the entrance of each arm, below the platform, a texture was attached. One texture indicated the presence of a water reward in the arm behind it, with the other texture indicating its absence. Thus, rats learned to perch at the edge of the gap in order to touch the texture with their long whiskers. The target behavior was to turn away on contact with a non-reward texture (Figure 1B (ii)) and to cross for a reward texture (Figure 1B (iii)). The trial ended when the rat entered one of the arms (Figure 1B (iv)). The entered arm was the start arm for the next trial. A computer pseudo-randomly chose the new reward arm, and the texture positions were changed accordingly.

On trials when the rat touched and then rejected the first texture, its encounter with the second texture was potentially prejudiced by prior knowledge. Therefore, on each trial, we examined behavioral and neuronal data only from primary encounters.

In this task, eight rats reached a stable performance (>75% correct on three successive sessions).

*The 1-arm task.* In this task, the rat remained in one arm of the maze, and on a given trial, only one texture was present, mounted on the central platform across the gap. The rat perched on the front edge of the rectangular platform and extended itself forward to contact the discriminandum with its whiskers (Figure 1A (i)). After palpating the texture, the rat withdrew and approached either the left or right drinking spout mounted near the platform (Figure 1A (ii)). The texture identity indicated the correct side to choose. Rats were trained with a fixed association (e.g., turn left on rough, right on smooth). Only if it approached the correct drinking spout was the rat given a water reward (0.2 ml); for an incorrect choice, it received no water. The next trial started as soon as the high-speed video was saved on the computer, a delay of about 10 s. Between trials, the discriminanda's mount was turned about its vertical axis by a computer-controlled stepping motor. This allowed for quick, randomized, and automated switching between discriminanda.

The session lasted until the rat failed to return to the discriminandum, signifying it was saturated.

In this task, six rats reached stable (>75%) performance.

### Video recording and analysis.

*Recording of films.* An overhead camera (Panasonic) recorded the rat's movements at 25 frames/s. Vibrissal motion and contact was recorded by high-speed (1,000 fps, 512 × 512 pixels) digital video cameras (Motionpro 2000; Redlake) triggered by a light sensor positioned near the discriminanda. Backlight illumination for the high-speed films came from a 12 × 12 array of LEDs emitting IR light at wavelength 880 nm (AOS technologies) which strobed in synchrony with individual video frames. Films were usually 1 s long.

*Analysis of films.* Films were analyzed off-line. For touch analysis, we extracted touch times (onset–offset) of the whiskers that were in the recorded neuronal clusters' receptive field. For a touch to be counted, the whisker had to be in contact with the texture plate and show a visible bending due to contact.

Moreover, we extracted the moment of choice. This was the time when the rat's behavior changed in a way indicating to the observer that it was about to cross or withdraw or otherwise stop examining the texture.

Independent analysis by different observers showed choice time deviations of just a few milliseconds.

Trials in which whisker touches could not be discerned were excluded from touch-based analysis, and trials in which the moment of choice could not be extracted were discarded.

### Surgery.

After reaching a performance of more than 75% correct on three consecutive sessions, rats were anaesthetized with a mixture of Zoletil (30 mg/kg) and Xylazine (5 mg/kg) delivered intraperitoneally. Small screws were fixed in the skull as a support for dental cement. One of the screws served as a ground electrode. A craniotomy was then made over barrel cortex, centered 2.8 mm posterior to bregma and 5.8 mm lateral to the midline [45]. Dura mater was left intact and covered with biocompatible silicon (KwikSil; World Precision Instruments). An eight-electrode drive (Neuralynx) or a 14-electrode drive (Kopf) was positioned above the craniotomy and attached by phosphate dental cement. Rats were given the antibiotic enrofloxacin (Baytril; 5 mg/kg delivered through the water bottle) and the analgesic caprofen (Rimadyl; 2.5 mg/kg, subcutaneous injection) for a week after surgery. For 10 d after surgery, they had unlimited access to water and food. Recording sessions in the apparatus began thereafter.

### Electrophysiological recordings.

Tungsten microelectrodes (Frederick Haer) were of 76-μm shaft diameter and impedance of 1–4 MΩ; they were advanced individually by rotation of a screw in the drive. At a depth of about 600 μm, it became possible to distinguish action potential waveforms evoked by manual whisker stimulation. Data reported here came from recordings at depths of 600–850 μm, as measured by the microdrive. The depth reading, together with the short response latencies (around 5 ms, see Figure 6) and the small receptive fields (1–2 whiskers; see [46,47]), indicate an electrode position in layer 4. After passing through a unity-gain head stage (Neuralynx), signals were transmitted through a cable to digitally programmable amplifiers (Cheetah Data Acquisition system; Neuralynx). The spike signals were amplified by a factor of 1,000–5,000, bandpass filtered between 600 Hz and 6 kHz, and digitized at 32 kHz; events that reached a user-set threshold were recorded for 1 ms (250 μs before voltage peak and 750 μs after peak). Spikes were sorted off-line on the basis of the amplitude and principal components by means of semiautomatic clustering algorithms (BBClust, written by P. Lipa, University of Arizona, Tucson, Arizona; and KlustaKwik, written by K. D. Harris, Rutgers University, Newark, New Jersey). The resulting classification was corrected and refined manually with MClust software (written by A. D. Redish, University of Minnesota, Minneapolis, Minnesota). Most electrodes yielded a multiunit neuronal cluster (*n* = 77), but in some cases (*n* = 8), we could isolate single units with a pronounced refractory period. Only neuronal clusters with stable waveform and firing rate over the course of a session were considered in the analysis.

For each cluster, the receptive field was mapped by manual stimulation of vibrissae. Nonresponsive clusters or clusters whose receptive field contained whiskers that were cut or were not used by the rat in the task were discarded.

To measure local field potentials in rat 10, a copy of the signal from an electrode that was used for the recording of spikes was amplified by a factor of 1,000, bandpass filtered between 1 Hz and 400 Hz, and digitized at 8 kHz. The receptive field of the neuronal clusters that were recorded from this electrode contained the whiskers C2 and C3. The reference electrode for these recordings was the bundle of steel guide tubes resting on the brain, above the recording site.

Electrode positions were frequently adjusted from one day to the next. Data recorded in different recording sessions from the same electrode, even if it was not moved, were always analyzed as separate clusters. This is because there was no proof that the same neuronal clusters were present at one electrode from day to day. This is a conservative assumption because, in the event that one cluster recorded on separate days was incorrectly evaluated as two clusters (two data points), this is equivalent to weighting a cluster by the number of days on which it was observed. Thus the differences in overall firing rate between rough and smooth texture contacts could not be created by the fracturing of data.

### Analysis of neuronal responses.

Quantitative analysis of neuronal coding of texture required the conjunction of many conditions: quality of the recorded neuronal activity, the rat's use of the whiskers that comprised the receptive fields of the neuronal clusters, reliable behavioral performance, and a sufficient number of trials per session and sessions per rat. Out of 14 rats trained in the tasks described above, electrodes were implanted successfully in ten animals. However, only one in the 3-arm task and two in the 1-arm task were able to satisfy the set of conditions stated above. These data are presented in Results. No selection of data was made a posteriori based on the strength or presence of texture coding.

*Weighted averaging of whisker contact responses.* To calculate the texture index *T* during whisker contact (see Equation 2), for each neuronal cluster, average firing rates were calculated from all contacts that the whisker(s) in the receptive fields of that cluster made in a session. As the total cumulative time of whisker contact varied significantly between clusters, the global average *T* across clusters was calculated with weights derived from each cluster's cumulative contact time. To accurately reflect the influence of rough and smooth contact time, the weight *w_cluster_* used was the inverse sum of the cumulative rough and smooth contact times *w_r_* and*w_s_*:

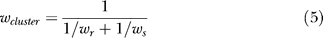



*Analysis of mutual information.* Three variables were of interest on each trial: discriminandum texture (rough or smooth), neuronal activity, and behavior of the animal. Mutual information proved to be a concise method for exploring their correlations. In general, the information that the neuronal response conveys about the stimulus can be quantified by Shannon's Mutual Information [16,17], hereafter simplified as “information,” as follows:

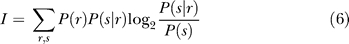
where *I* is the information, *P*(*s*) is the probability of presentation of stimulus *s* (rough or smooth), *P*(*s*|*r*) is the posterior probability of *s*, given observation of response *r*, and *P*(*r*) is the probability of response *r* unconditional on the stimulus. Information measured by Equation 6 quantifies how well an ideal observer can discriminate between members of a stimulus set based on the neuronal responses of a single trial [18].

For each trial, neuronal response was defined as the number of spikes during variable time windows prior to the moment of choice.

The conditional probabilities in the above formulas are not known a priori and must be estimated empirically from a limited number, *N*, of experimental trials for each stimulus. For some recordings in our dataset, *N* could be as low as 12. Limited sampling of response probabilities can lead to an upward bias in the estimate of mutual information [48–52]. The bias magnitude depends on the number of trials per stimulus or behavior: as *N* increases, the estimated probabilities become more accurate, and the bias decreases. An approximate expression for the bias has been formulated [49] and can be subtracted from the raw information of Equation 6, provided that *N* is at least two to four times greater than the number of different possible response classes, *R* [49,53]. To reduce bias, we therefore reduced the dimensionality of the response space *R* by subdividing the firing rates into only two classes, high firing rate and low firing rate, separated by the median value. This meant that there were the same number of observations of each of the two response classes. We then applied the bias subtraction procedure.

*Analysis of incorrect trials.* When analyzing trials in which the animal's behavior was incorrect, the goal was to find whether the stimulus signal was altered with respect to correct trials; it could be weakened, at random, reversed, or unchanged. As explained in Results, this was done indirectly by comparing correct trials to a randomly chosen, repetitively sampled subset of the same size, potentially containing incorrect trials. Any way in which the incorrect trials differed from the correct trials was reflected in the random trials. Without proof, we conjecture that the statistical power of a test aimed at detecting a correct–incorrect difference in this indirect way is equal to the direct way, where applicable.

*Statistical tests of significance.* To test an effect of texture on a given statistic (texture index, information, etc.), a permutation test was used. Thus, no assumptions were made on the kind of distributions involved. The null hypothesis *H_0_* was that texture had no influence on the statistic. Thus, for any given recording session, the trials' texture labels were scrambled, leaving the total number of rough and smooth trials unchanged. The statistic was calculated using the scrambled labels. This procedure was repeated 1,000 times, and the real value of the statistic was ranked in the distribution of scrambled values, yielding a *p*-value.

To prove an effect of the rat's behavior, i.e., of trials being correct or incorrect, an analogous procedure was used, scrambling the correctness labels instead of the texture labels. As explained in the previous section, correct trials were not compared directly to incorrect trials, but rather to randomly chosen, mixed subsets of the same size as the correct trials. For the *p*-value, the statistic was calculated on all subsets, and the correct-only value ranked in the values of 1,000 random sets.

For non-negative statistics (information), all tests were one-tailed.

For statistics taking on negative and positive values, tests were two-tailed. An exception was the texture index *T*, because previous studies [9] indicated that *T* > 0, i.e., there would be more spikes during rough than during smooth trials.

When averaging across neuronal clusters that were recorded simultaneously, care was taken to apply the same texture permutation to them, thus preserving noise correlation [54].

## Supporting Information

Video S1 High-Speed Film, Top View Filmed at 1,000 frames/s, shown slowed down by a factor of 20. Clicks in the soundtrack correspond to spikes of a neuronal cluster responsive to whisker C2, the third long whisker counting from the top.
(695 KB WMV)

Video S2 High-Speed Film, Side View Same as Video S1, but with a different angle of view.
(1.03 MB WMV)

Video S3 Overhead View of 1-Arm Task Three trials of the 1-arm task. The film is slowed down by a factor of 4. Note the two mobile parts: on the rotating platform facing the rat, the textures are mounted. The rotating object in front of the rat's platform is a paw support. It is retracted when the texture is changed to ensure that the rat cannot touch the discriminandum until it reaches its final position. The drinking wells are near the bright LED spots, on either side of the platform. The bright squares that are visible in the top left of the frame are LED matrices for the high speed cameras.
(270 KB WMV)

## Abbreviations

LED: light-emitting diode
LFP: local field potential
PCTH: peri-contact time histogram

## References

[pbio-0050305-b001] Gamzu E, Ahissar E (2001). Importance of temporal cues for tactile spatial- frequency discrimination. J Neurosci.

[pbio-0050305-b002] Kleinfeld D, Ahissar E, Diamond ME (2006). Active sensation: insights from the rodent vibrissa sensorimotor system. Curr Opin Neurobiol.

[pbio-0050305-b003] Shuler MG, Krupa DJ, Nicolelis MA (2001). Bilateral integration of whisker information in the primary somatosensory cortex of rats. J Neurosci.

[pbio-0050305-b004] Carvell GE, Simons DJ (1990). Biometric analyses of vibrissal tactile discrimination in the rat. J Neurosci.

[pbio-0050305-b005] Knutsen PM, Derdikman D, Ahissar E (2005). Tracking whisker and head movements in unrestrained behaving rodents. J Neurophysiol.

[pbio-0050305-b006] Schiffman HR, Lore R, Passafiume J, Neeb R (1970). Role of vibrissae for depth perception in the rat (Rattus norvegicus). Anim Behav.

[pbio-0050305-b007] Woolsey TA, der Loos HV (1970). The structural organization of layer iv in the somatosensory region (si) of mouse cerebral cortex. the description of a cortical field composed of discrete cytoarchitectonic units. Brain Res.

[pbio-0050305-b008] Arabzadeh E, Zorzin E, Diamond ME (2005). Neuronal encoding of texture in the whisker sensory pathway. PLoS Biol.

[pbio-0050305-b009] Arabzadeh E, Panzeri S, Diamond ME (2006). Deciphering the spike train of a sensory neuron: counts and temporal patterns in the rat whisker pathway. J Neurosci.

[pbio-0050305-b010] Sellien H, Eshenroder DS, Ebner FF (2005). Comparison of bilateral whisker movement in freely exploring and head-fixed adult rats. Somatosens Mot Res.

[pbio-0050305-b011] Mehta SB, Whitmer D, Figueroa R, Williams BA, Kleinfeld D (2007). Active spatial perception in the vibrissa scanning sensorimotor system. PLoS Biol.

[pbio-0050305-b012] Crochet S, Petersen CCH (2006). Correlating whisker behavior with membrane potential in barrel cortex of awake mice. Nat Neurosci.

[pbio-0050305-b013] Petersen RS, Diamond ME (2000). Spatial-temporal distribution of whisker-evoked activity in rat somatosensory cortex and the coding of stimulus location. J Neurosci.

[pbio-0050305-b014] Frien A, Eckhorn R (2000). Functional coupling shows stronger stimulus dependency for fast oscillations than for low-frequency components in striate cortex of awake monkey. Eur J Neurosci.

[pbio-0050305-b015] Hentschke H, Haiss F, Schwarz C (2006). Central signals rapidly switch tactile processing in rat barrel cortex during whisker movements. Cereb Cortex.

[pbio-0050305-b016] Shannon CE (1948). A mathematical theory of communication. Bell Syst Tech J.

[pbio-0050305-b017] Cover T, Thomas J (1991). Elements of information theory.

[pbio-0050305-b018] Rieke F, Warland D, van Steveninck RR, Bialek W (1997). Spikes: exploring the neural code.

[pbio-0050305-b019] Guic-Robles E, Valdivieso C, Guajardo G (1989). Rats can learn a roughness discrimination using only their vibrissal system. Behav Brain Res.

[pbio-0050305-b020] Derdikman D, Yu C, Haidarliu S, Bagdasarian K, Arieli A (2006). Layer-specific touch-dependent facilitation and depression in the somatosensory cortex during active whisking. J Neurosci.

[pbio-0050305-b021] Ferezou I, Bolea S, Petersen CCH (2006). Visualizing the cortical representation of whisker touch: voltage-sensitive dye imaging in freely moving mice. Neuron.

[pbio-0050305-b022] Darian-Smith I, Sugitani M, Heywood J, Karita K, Goodwin A (1982). Touching textured surfaces: cells in somatosensory cortex respond both to finger movement and to surface features. Science.

[pbio-0050305-b023] Sinclair RJ, Burton H (1991). Neuronal activity in the primary somatosensory cortex in monkeys (Macaca mulatta) during active touch of textured surface gratings: responses to groove width, applied force, and velocity of motion. J Neurophysiol.

[pbio-0050305-b024] Tremblay F, Ageranioti-Bélanger SA, Chapman CE (1996). Cortical mechanisms underlying tactile discrimination in the monkey. I. Role of primary somatosensory cortex in passive texture discrimination. J Neurophysiol.

[pbio-0050305-b025] Panzeri S, Schultz SR (2001). A unified approach to the study of temporal, correlational, and rate coding. Neural Comput.

[pbio-0050305-b026] Panzeri S, Petersen RS, Schultz SR, Lebedev M, Diamond ME (2001). The role of spike timing in the coding of stimulus location in rat somatosensory cortex. Neuron.

[pbio-0050305-b027] Petersen RS, Panzeri S, Diamond ME (2001). Population coding of stimulus location in rat somatosensory cortex. Neuron.

[pbio-0050305-b028] Norena A, Eggermont JJ (2002). Comparison between local field potentials and unit cluster activity in primary auditory cortex and anterior auditory field in the cat. Hear Res.

[pbio-0050305-b029] Gray CM, Maldonado PE, Wilson M, McNaughton B (1995). Tetrodes markedly improve the reliability and yield of multiple single-unit isolation from multi-unit recordings in cat striate cortex. J Neurosci Meth.

[pbio-0050305-b030] Hipp J, Arabzadeh E, Zorzin E, Conradt J, Kayser C (2006). Texture signals in whisker vibrations. J Neurophysiol.

[pbio-0050305-b031] Albarracín AL, Farfán FD, Felice CJ, Décima EE (2006). Texture discrimination and multi-unit recording in the rat vibrissal nerve. BMC Neurosci.

[pbio-0050305-b032] Moore C, Andermann M, Ebner FF (2005). The vibrissa resonance hypothesis. Neural plasticity in adult somatosensory-motor systems.

[pbio-0050305-b033] Guic-Robles E, Jenkins WM, Bravo H (1992). Vibrissal roughness discrimination is barrelcortex-dependent. Behav Brain Res.

[pbio-0050305-b034] Prigg T, Goldreich D, Carvell GE, Simons DJ (2002). Texture discrimination and unit recordings in the rat whisker/barrel system. Physiol Behav.

[pbio-0050305-b035] Szwed M, Bagdasarian K, Blumenfeld B, Barak O, Derdikman D (2006). Responses of trigeminal ganglion neurons to the radial distance of contact during active vibrissal touch. J Neurophysiol.

[pbio-0050305-b036] Fee MS, Mitra PP, Kleinfeld D (1997). Central versus peripheral determinants of patterned spike activity in rat vibrissa cortex during whisking. J Neurophysiol.

[pbio-0050305-b037] O'Connor SM, Berg RW, Kleinfeld D (2002). Coherent electrical activity between vibrissa sensory areas of cerebellum and neocortex is enhanced during free whisking. J Neurophysiol.

[pbio-0050305-b038] Ganguly K, Kleinfeld D (2004). Goal-directed whisking increases phase-locking between vibrissa movement and electrical activity in primary sensory cortex in rat. Proc Natl Acad Sci U S A.

[pbio-0050305-b039] Ahrens KF, Kleinfeld D (2004). Current flow in vibrissa motor cortex can phase-lock with exploratory rhythmic whisking in rat. J Neurophysiol.

[pbio-0050305-b040] Szwed M, Bagdasarian K, Ahissar E (2003). Encoding of vibrissal active touch. Neuron.

[pbio-0050305-b041] Salinas E, Hernandez A, Zainos A, Romo R (2000). Periodicity and firing rate as candidate neural codes for the frequency of vibrotactile stimuli. J Neurosci.

[pbio-0050305-b042] Rolls ET, Critchley HD, Treves A (1996). Representation of olfactory information in the primate orbitofrontal cortex. J Neurophysiol.

[pbio-0050305-b043] Gochin PM, Colombo M, Dorfman GA, Gerstein GL, Gross CG (1994). Neural ensemble coding in inferior temporal cortex. J Neurophysiol.

[pbio-0050305-b044] Grunewald A, Linden JF, Andersen RA (1999). Responses to auditory stimuli in macaque lateral intraparietal area. I. Effects of training. J Neurophysiol.

[pbio-0050305-b045] Paxinos G, Watson C (2007). The rat brain in stereotaxic coordinates. 4th Edition.

[pbio-0050305-b046] Brecht M, Sakmann B (2002). Dynamic representation of whisker deflection by synaptic potentials in spiny stellate and pyramidal cells in the barrels and septa of layer 4 rat somatosensory cortex. J Physiol.

[pbio-0050305-b047] Chapin JK, Lin CS (1984). Mapping the body representation in the SI cortex of anesthetized and awake rats. J Comp Neurol.

[pbio-0050305-b048] Optican LM, Gawne TJ, Richmond BJ, Joseph PJ (1991). Unbiased measures of transmitted information and channel capacity from multivariate neuronal data. Biol Cybern.

[pbio-0050305-b049] Panzeri S, Treves A (1996). Analytical estimates of limited sampling biases in different information measures. Network.

[pbio-0050305-b050] Golomb D, Hertz J, Panzeri S, Treves A, Richmond B (1997). How well can we estimate the information carried in neuronal responses from limited samples?. Neural Comput.

[pbio-0050305-b051] Victor JD (2000). Asymptotic bias in information estimates and the exponential (bell) polynomials. Neural Comput.

[pbio-0050305-b052] Paninski L (2003). Convergence properties of three spike-triggered analysis techniques. Network.

[pbio-0050305-b053] Pola G, Thiele A, Hoffmann KP, Panzeri S (2003). An exact method to quantify the information transmitted by different mechanisms of correlational coding. Network.

[pbio-0050305-b054] Oram MW, Földiák P, Perrett DI, Sengpiel F (1998). The ‘ideal homunculus': decoding neural population signals. Trends Neurosci.

